# Radiology Review - 6^th^ Edition

**Published:** 2008-02

**Authors:** Pradeep Krishnan

**Affiliations:** Department Of Radiology, Wellspring-Jankharia Imaging, Bhaveshwar Vihar, 383 Sardar V P Rd, Mumbai - 400 004 Email: pradeep@jankharia.com

## Review-2

This is the sixth edition of the *Radiology Review Manual* since its first edition published almost two decades. The book has been a handy reference guide for radiology residents, fellows and practicing radiologists. It summarises the basic and pathology of multiple systems from head to foot.

The contents are organized into major sections, which include anatomy and radiological patterns for differential diagnosis in the beginning of each chapter with detailed information on the disease entity towards the end of chapter. A great deal of effort has been put to present differential diagnosis in an organized manner, although it could have been made more logically. The basic anatomy and pathology has been covered in outline form. The book provides the key concepts and synopsis of information essential for practicing radiologists and students alike, but it is not meant for an in-depth reference on any given topic. Its format enables users to refer to it easily in educational as well as clinical settings.

However, the book gives the impression of a slightly cluttered appearance with overuse of arrows and notations. The mnemonics are a turnoff, although some radiology residents would find it useful for their board exams. The book could include suggested readings at the end of each chapter.

Although not meant to be all-inclusive, the book is fairly comprehensive and provides concise package of facts about a given disease for quick and easy assess, making it a very efficient resource for library reference.

